# One-Pot Production of RNA in High Yield and Purity Through Cleaving Tandem Transcripts

**DOI:** 10.3390/molecules25051142

**Published:** 2020-03-04

**Authors:** Hannes Feyrer, Raluca Munteanu, Lorenzo Baronti, Katja Petzold

**Affiliations:** Department of Medical Biochemistry and Biophysics, Karolinska Institutet, 171 77 Stockholm, Sweden

**Keywords:** RNA preparation, *in vitro* transcription, T7 polymerase, RNase H, tandem repeats, short RNAs

## Abstract

There is an increasing demand for efficient and robust production of short RNA molecules in both pharmaceutics and research. A standard method is *in vitro* transcription by T7 RNA polymerase. This method is sequence-dependent on efficiency and is limited to products longer than ~12 nucleotides. Additionally, the native initiation sequence is required to achieve high yields, putting a strain on sequence variability. Deviations from this sequence can lead to side products, requiring laborious purification, further decreasing yield. We here present transcribing tandem repeats of the target RNA sequence followed by site-specific cleavage to obtain RNA in high purity and yield. This approach makes use of a plasmid DNA template and RNase H-directed cleavage of the transcript. The method is simpler and faster than previous protocols, as it can be performed as one pot synthesis and provides at the same time higher yields of RNA.

## 1. Introduction

Ribonucleic acid (RNA) is one of the main building blocks of life. It is not only accepted as being the first self-replicating precursor of all life, but still maintains an essential role in all living organisms. Apart from encoding protein sequence as messenger RNA, it also forms major regulatory pathways between the genome and the proteome through a vast number of RNA classes [1,2], like microRNA (miRNA) [3], piRNA [4] or long non-coding RNA [5], and performs essential catalytical processes [6], such as those in the ribosome [7] and the spliceosome [8]. RNA has received strong attention in the medical field through milestone discoveries in the fields of RNA interference [9] or CRISPR-Cas9 gene editing [10,11]. With the expanding potential for RNA-based therapeutics, there is a need for reliable and efficient production of RNA in milligram amounts or more, both for medical application and basic research [12,13]. 

Commercial RNA is often produced through solid-phase synthesis. This is limited to short sequences (~100 nucleotides (nt)) and is comparatively costly, but offers the possibility to incorporate chemical modifications, which can facilitate delivery and increase stability and activity in cells [14,15]. While modified RNAs are most widely used for downregulation or therapeutic applications, some publications are advocating the use of native RNA [16]. 

The method of choice for in-house production of non-modified RNA samples is *in vitro* transcription (IVT) using RNA polymerase from bacteriophage T7 (T7RNAP) [17,18]. While this is a versatile and well-performing enzyme in general, it exhibits some issues with the transcription of certain templates, like short constructs (15–50 nt) or those with pyrimidine-rich start sequences. These issues have been well characterized in the past, such as 5′- and 3′-inhomogeneity and low yield, due to suboptimal initiation sequences [19,20,21,22,23]. The full-length construct has to be purified from shorter and longer molecules, usually using polyacrylamide gel excision, ion-exchange or size-exclusion chromatography [24,25,26]. Issues with transcription have been addressed and solved to a large extent, but many of these solutions are not compatible with certain applications or do not increase the yield of difficult constructs. The issues pertain, in particular, for short constructs ~20 nt with a pyrimidine-rich initiation site and the need of defined ends, such as miRNAs [27]. 

A common method to increase T7RNAP processivity and termini homogeneity is an alteration of the DNA template. 5′-inhomogeneity can be reduced by addition of G-rich initiation-sequences directly after the T7 promotor. Furthermore, 2′-methoxylation of the final two 5′-nucleotides of the DNA template should suppress the addition of non-templated nucleotides on the 3′-end of the RNA product [28]. Yield and transcript length homogeneity have shown to be somewhat sequence-dependent and can be improved by optimizing the ratios of reagents in the transcription reaction [29]. T7RNAP has been engineered via mutagenesis to improve yield and promiscuity for nucleotide incorporation [30]. While the latter two have no impact on the final product, extending the initiation sequence can change structure and function [29].

Another means of decreasing 5′- and 3′-inhomogeneity is the incorporation of cis-acting ribozymes that self-cleave after transcription, leaving well-defined 5′- and 3′-ends [31]. However, the side products created have a one-to-one ratio to the target, which can hamper the purification. More importantly, the commonly used hammerhead ribozyme leaves two nucleotides after the target sequence, thus, extending the desired product and altering its sequence. Furthermore, ribozymes produce 2′,3′-cyclic phosphate and 5′-OH termini. This can be a major impairment to the molecule’s function, like it has been shown for 5′-dephosphorylated miRNAs [32]. 

RNase H has been employed to replace ribozymes for the removal of initiation sites, or excise labelled fragments [32,33]. *Escherichia coli* RNase H cleaves the RNA phosphodiester bond in an RNA-DNA hybrid duplex opposite the DNA’s 5′-end, leaving native 3′-OH and 5′-monophosphate termini [34,35,36]. The disadvantage of this method is the requirement for the additional enzyme and a DNA splint (or modified oligo) to guide the enzyme to the cleavage site.

Wang et al. presented a method for *in vitro* production of miRNA that is based on promoter-free rolling circle transcription from a short, single-stranded, circular DNA to produce a tandem repeat transcript, which is successively cleaved by RNase H [37]. A similar method was used for the generation of RNA nanoparticles for siRNA delivery into cells [38]. 

**Figure 1:**
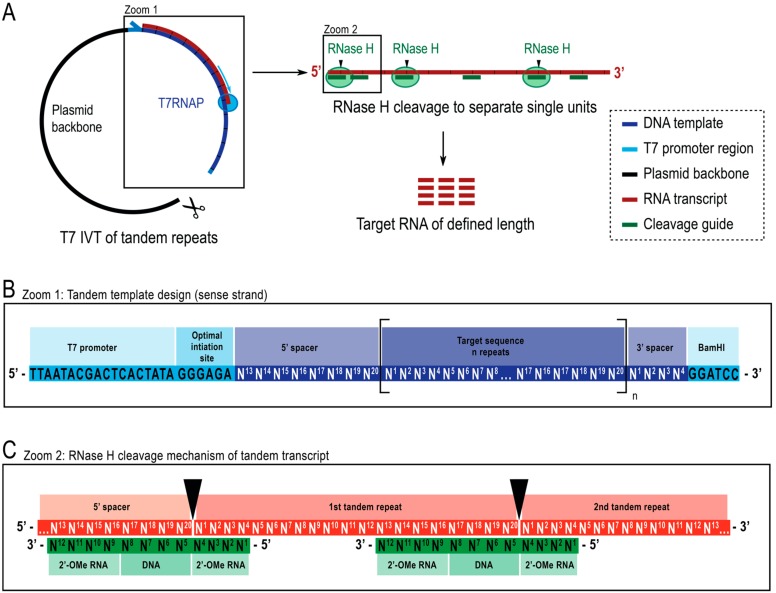
Schematic representation of the reported protocol. (**A**): (left) Tandem transcription from a linearized plasmid template with T7 RNA polymerase (T7RNAP) and (right) successive cleavage of the transcript to target length RNA by RNase H, directed by a chimeric DNA guide. (**B**): Detailed schematic of the tandem template, which starts with the viral T7RNAP promoter, an initiation sequence. The target sequence (dark blue, example here is 20 nt length) is repeated n number of times. The repeats flanked by a 5′- and 3′-spacer sequences consisting of the last eight and first four nucleotides respectively to allow for removal of the initiation and restriction sequences from the first and last repeat unit. (**C**): Hybridization of the tandem transcript (red) and the chimeric cleavage guides (green). RNase H cleaves the RNA opposite the DNA 5′-end. The 2′-OMe RNA flanks increase specificity by enhancing the binding affinity of cleavage guide to the target RNA.

Herein we report a simple and cost-effective strategy for the enzymatic production of short, non-modified RNAs in high yield and purity. We use a plasmid encoding tandem repeats of the target RNA sequence. The resulting transcript is cleaved with RNase H guided by a chimeric cleavage splint to produce single repeat units of precise length. The resulting pool of cleaved RNAs is highly homogenous, simplifying downstream purification and improving subsequent yield (Figure 1).

## 2. Results

We here present a one-pot method that facilitates enzymatic RNA production by transcribing from a plasmid encoding a repetitive template and thereafter site-specifically separating the tandem repeats with RNase H cleavage guided by a short chimeric oligo to release single target RNAs (Figure 1). These short RNAs are then purified with one single ion-exchange HPLC step. The obtained RNA yields have been determined with an external HPLC standard curve, and purity and integrity of the RNA constructs have been validated with denaturing polyacrylamide gel electrophoresis (PAGE). 

We tested this method on six different constructs, one of 20 nt, four of 22 nt, and one of 75 nt length. None of them starts with a GG sequence, and are therefore, poor candidates for conventional IVT.

### 2.1. Template Design

For the transcription of tandem repeats, we generated a template consisting of a T7 promotor (5′ – TAA TAC GAC TCA CTA TA – 3′) followed by an optimal initiation sequence (5′ – GGG AGA – 3′) (Figure 1). These are followed by a number, as high as feasible for DNA template production, of tandem repeats of the target RNA sequence. Adding the optimal initiation sequence leads to the first repeat being slightly longer. Additionally, the last repeat could be extended by, e.g., residual sequences of a restriction site or a mere buffer against 3′-inhomogeneity of transcription. In constructs 1 and 6, we eliminate the additions by including spacer regions that act as recognition sites for the cleavage guide (Figure 1C). The 5′-spacer sequence consists of the last ~8 nt of the target, and the 3′-spacer sequence consists of the first ~4 nt of the target sequence (numbers are exemplary for a 12 nt long cleavage guides), to allow for cleavage. 

We chose plasmid DNA, as they are reliable templates, because a high number of repeats is possible, and maintenance is simple and well-established. We used a pUC19 vector as a plasmid backbone and purchased a 600 bp insert cloned into the vector (insert synthesis and subcloning performed by Genscript), which resulted in e.g. for construct 2: 26 repeats of a 22 nt target RNA after the T7 promoter (detailed information in SI). The cloning site was chosen so that a BamHI recognition site is within a few base pairs downstream of the insert for linearization. It can be problematic to verify the exact number of repeats in a construct by sequencing; however, the exact number of repeats is not strictly important for this approach, as it does not influence the result apart from the ratio of pure cleaved product to side-product.

### 2.2. T7 In Vitro Transcription

The transcription condition from the linearized plasmid of construct 2 has been optimized with published conditions as a starting point [39]. Interestingly, the optimal conditions (Supplementary Information Table S2) for the transcription reactions did not depend on the sequence of the repeat unit, as we expected it based on experience from IVT on short single-stranded DNA (ssDNA) templates. This can be explained by the use of long double-stranded templates with the same initiation sequence, which allows high processivity of T7RNAP. However, the overall yield (10–30%) did show sequence-dependency and could not be increased with further optimization of the reaction conditions. This can, however, be seen as an advantage, as time-consuming optimization of reaction conditions is normally an integral part of IVT for every new construct. 

**Figure 2:**
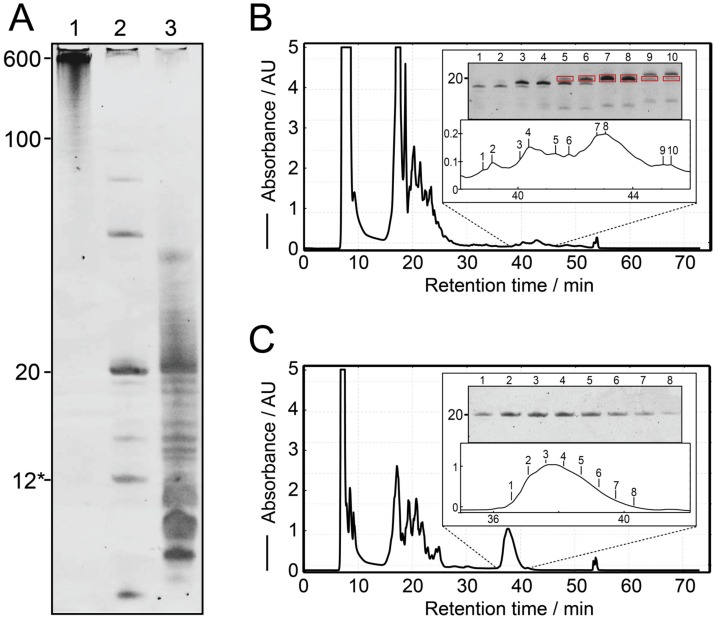
Proof of principle on construct 1. (**A**): Denaturing polyacrylamide gel showing the cleavage of a tandem transcript and comparison of transcription from a single-repeat ssDNA template. Lane 1: Tandem transcription of the construct from the linearized plasmid. Lane 2: Simultaneous RNase H cleavage during tandem transcription leading to the main product of 20 nt. The 12* label refers to the chimeric cleavage guide consisting of 4 DNA nucleotides and 8 2′-OMe nucleotides. Lane 3: IVT from a single-repeat template encoding construct 1 showing much higher levels of longer and shorter products. For comparison, the same amount of reaction has been loaded in all lanes. (**B**): Ion-exchange HPLC chromatogram for the 1 mL IVT from A (lane 3, construct 1*) and denaturing PAGE sampling the eluted peaks. The target peak overlaps with side products and is of low intensity. (**C**): Ion-exchange HPLC chromatogram for a 1 mL reaction of cleaved tandem transcription from A (lane 2, construct 1). The main signal is pure judging from denaturing PAGE, and of significantly larger intensity than from single-repeat template.

**Figure 3:**
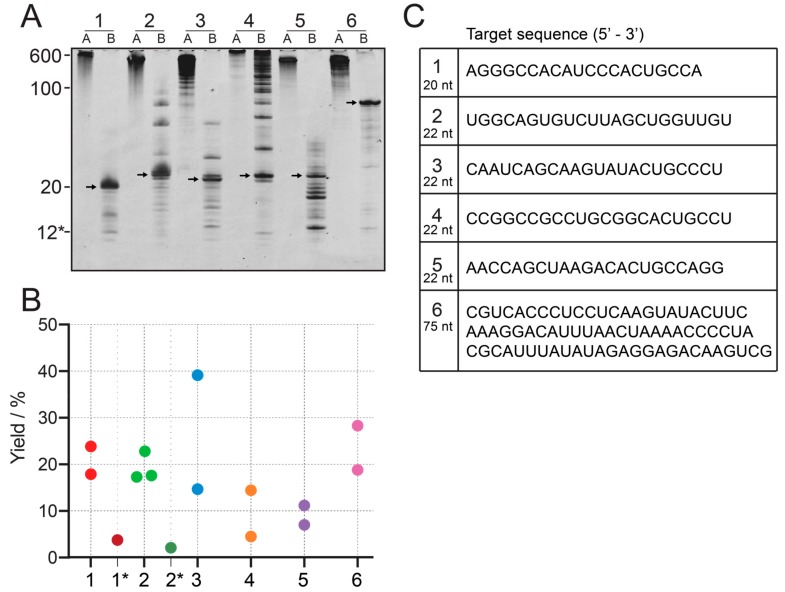
**Yield comparison between 6 different constructs.** (**A**): IVT (lanes A) and cleaved tandem transcription (lanes B) for six constructs. 1: 20 nt, 2–5: 22 nt, 6: 75 nt. To allow comparison between constructs, the same volume of reaction has been loaded onto all lanes. The 12* label refers to the chimeric cleavage guide of construct 1, consisting of 4 DNA nucleotides and 8 2′-OMe nucleotides. (**B**): Yield quantification from IE HPLC for the six constructs, including transcription from single-repeat template for construct 1 and 2, labeled 1* and 2* using an external standard curve. Individual points denote technical replicates. Numbers can be found in the Supplementary Information (Table S3). (**C**): RNA sequences of all six constructs.

As proof of principle, the IVT of construct 1 is shown in Figure 2A, lane 1. When the transcribed RNA was loaded onto a 20% denaturing PAGE, the main band of ~600 nt was the strongest, even though shorter transcripts can be observed. These shorter constructs, however, will still largely contribute to the final product, as they still contain a large number of intact target sequences, while imperfect sequences will be cleaved off. We found that Mg^2+^ concentrations above 40 mM inhibit the reaction for construct 2 and construct 3 (data not shown). The transcription reactions for all 6 constructs can be seen in Figure 3, in the respective lane A. They all show the main band around 600 nt, with the exception of the construct 4, which produces a longer band.

### 2.3. RNase H Cleavage Reaction

*E.coli* RNase H cleaves the RNA in an RNA-DNA duplex opposite the DNA’s 5′-end and shows improved specificity and efficiency if the DNA sequence is flanked by modified nucleotides with an enhanced binding affinity to RNA. For this purpose, 2′-OMe modifications are employed [36] (Figure 1C). We designed the cleavage guides to have 4 central DNA nucleotides flanked by 4 modified nucleotides on each site. It has been shown that longer flanks can increase efficiency and reduce side products [33].

In our experiments, RNase H readily produces single-repeat units of the target RNA in a separate cleavage reaction or even directly during the T7 IVT, allowing for a one-pot reaction (Figure 2A, lane 2). In comparison, the IVT from a single-repeat oligo is significantly weaker in intensity and shows more bands from shorter and longer products (Figure 2A, lane 3). The cleavage reaction went to completion for all constructs, but construct 4, which can be monitored by reduction of the high molecular weight bands in Figure 3A. We found that cleavage reactions were not complete after 16 h for construct 2 and 5. In this case, the annealing has been repeated, followed by the addition of additional RNase H (heating the sample to 95°C, cooling down and adding the initial amount of RNase H again). This generally helped to complete the reactions. Depending on transcription yield, the final concentration of the cleavage guide of 20 µM gives a ratio of 1:0.2 to 1:1 of cleavage sites on the tandem transcripts to cleavage guide in the sample. Previous protocols for the preparation of RNA samples used a ratio of 1:1.2 [34], 1:20 [33] and 1:600 [37]. An increase of cleavage efficiency using molar excess of cleavage guide, however, could not be confirmed by our work.

The RNA constructs created from cleavage of tandem transcripts are of very high purity after completion of the reaction. Unspecific cleavage was observed for some constructs, where the weaker n ± 1 band can be seen below the 22 nt target band (Figure 3A). Also, constructs without the previously mentioned 3′- and 5′-spacer regions (constructs 2, 3, 4 and 5) can show some longer constructs which are connected to the initiation site and restriction site and the beginning and end of the tandem region. For construct 4, the cleavage was not complete, and uncleaved transcript was still detectable. It should be noted, that the cleavage guide for construct 4 is 16 nt, instead of 12 nt. It carries a 6 nt flank of 2′-OMe modified nucleotides on both ends. This was done to increase affinity to the target site, because a second site with a similar sequence to the cleavage site is present in the transcript. Cleavage from the second site could not be detected, but instead, the cleavage reaction is not complete. For construct 5, a significant amount of shorter products can be observed.

*E.coli* RNase H shows unimpaired enzymatic activity within buffer systems of the T7 buffer and the commercial RNase H reaction buffer (NEB). This renders purification between T7 transcription and RNase H cleavage reactions obsolete and means, and they can be performed in the same vessel. Simultaneous and successive reactions showed no difference in yield and purity, but could shorten the overall time requirements.

### 2.4. Ion-Exchange HPLC Purification

Preparative HPLC can struggle to separate nucleic acids of similar length, due to their small relative difference in size and charge. Our group recently developed a protocol to purify milligrams of short RNA to a single-nucleotide resolution with ion-exchange HPLC [40]. Using the same method, we could purify the target RNA after RNase H cleavage. Due to the absence of similar-size RNA, milligrams of RNA could be purified with a single injection, minimizing working hours and yield loss. Sampling single fractions to ensure the highest purity is not necessary, but can improve purity if many unspecific cleavage products are detected by gel electrophoresis prior to HPLC purification.

Figure 2 shows ion-exchange HPLC chromatograms for construct 1 from a single-repeat template (Figure 2B, lane 3 in Figure 2A) and tandem template (Figure 2C, lane 2 in Figure 2A) in comparison. Denaturing PAGE of the peaks from a single-repeat template (Figure 2B) shows the target bands and indicates the fractions containing the target RNA. Only the purest fractions can be pooled to obtain a pure sample (fractions 7 and 8). A lot of transcribed material, including target-length RNA, has to be discarded or re-purified (e.g., fractions 5, 6, and 9). Oppositely, using the tandem transcript cleavage method (Figure 2C), the entire volume of the chromatographic peak can be pooled to give a pure final sample. This allows for a simpler and faster purification method, as more material can be loaded without limitation imposed by the chromatographic resolution [40]. 

### 2.5. Yield Quantification 

Reaction yield was quantified with HPLC using an external standard of known amounts of construct 2. A standard curve of samples containing 149.8, 74.9, 37.4, 18.7 and 9.3 nmol of construct 2 was created, confirmed by UV spectroscopy, to allow quantification of HPLC analyte samples after normalization of molar extinction coefficients (Supplementary Information Figure S1). Yields of example reactions of 1 mL cleaved tandem transcription reaction is shown in Figure 3B. The yields from cleaved tandem IVT are on average 20% (~7-fold improvement for construct 1 and 2), which is significantly higher than the example of single-repeat transcription of the construct 1* (initiation site starting with adenosine) and 2* (initiation site starting with uridine). To sample variation in yield, data has been obtained for 2 or 3 technical replicates. Construct 3 shows high variation between the two replicates, which cannot be explained at this point.

### 2.6. Differences Between Small Scale and Large Scale Reactions

When scaling up, we usually found no correlation of yield to reaction scale. However, a difference in generation of inorganic pyrophosphate precipitates during transcription and cleavage efficiency between small scale (20–50 µL) and large scale (1–40 mL) reactions was observed. Precipitation during T7 transcription reactions is attributed to aggregation of pyrophosphate with magnesium ions and nucleic acids [41]. While there is usually no precipitation observed in small scale reactions, it is abundant in large scale reactions. This can be an issue, because it removes RNA from solution and makes it inaccessible to RNase H cleavage. Addition of inorganic pyrophosphatase (IPPase) prevents the formation of such precipitates [41]. After microwave heating, precipitates form again, which could again be degraded with the addition of IPPase together with RNase H. Furthermore, the duration for cleavage completion was found to be longer for large scale reactions, despite accurate scale-up of all components. At this point, there are no explanations for this behavior. 

## 3. Discussion

### 3.1. Large Scale Production of RNA from Repetitive Templates

The protocol we present here aims at maximizing the yield of synthesized RNA and simplifying the purification of the product. In synthetic chemistry, the yield is defined as the ratio of obtained pure product to input of limiting reagent. In contrast to this, biochemical publications on RNA production often report on fold amplification compared to the input DNA template, often in units of mass [18]. The DNA template, however, is not consumed in the reaction, and hence, not the limiting reagent. Instead, the nucleotide triphosphates, which form the growing RNA chain, are the limiting compound and have to, therefore, be used as the reference when calculating the molar yield. This is especially important when aiming for pure RNA, because RNA products of similar lengths, which do not contribute to the final product, have to be discarded and seen as a side product of the reaction, leading to a reduction in yield. 

Compared to state-of-the-art protocols [26], our method results in increased yield because of the optimal initiation site, the tandem design of the transcript, intermediate RNase H cleavage and the resulting facilitated purification.

The optimal initiation site (5′ – GGG AGA – 3′ ) increases the overall production of RNA through facilitating the binding and altering the processivity of the T7 RNA polymerase [19]. Addition of such a guanosine-rich initiation site is common practice in the field to increase the RNA yield. However, a separate cleavage step is required to remove the initiation sequence if it interferes with the biological function [31,34]. In our protocol, the design of the template allows us to remove the initiation site simultaneously with the cleavage of the tandem transcript to produce single units of target RNA (Figure 1). As a further advantage, this removes the need for optimization of transcription conditions, as is typically necessary for single-repeat transcripts with varying initiation sites. Furthermore, the design of the template increases the yield, because no longer or shorter sequences can be transcribed. The step determining the purity of the final RNA molecules is the RNase H cleavage, which we have shown to be much more precise than the single-repeat transcription of a short RNA (Figure 2A). Additionally, every initiation event does not only lead to one RNA molecule, but to as many copies as are encoded in the template, exploiting the high processivity of T7 RNA polymerase most optimally. For this reason, templates containing a high number of tandem units are favored.

The increased starting purity of our samples simplifies purification and allows us to retain more of the original synthesized product in the final sample, therefore, increasing the yield. Typically, the purification of single copy transcripts was hampered by side products of similar length in high concentration, where their removal is a challenge in preparative chromatography or gel electrophoresis methods ([24], Figure 2B,C). Because the method we present here increases the starting purity significantly, a fast and simple purification method is sufficient to remove possibly uncleaved target dimers (twice the target size), and the cleavage splint (at least half the target size).

Another factor for yield loss comes from the mere number of handling steps of the sample, such as buffer-exchange or change of vessel, where RNA is lost. This issue is especially present for the annealing step of large volumes, as the sample requires heating to ~95 °C. The annealing process in the presence of magnesium needs to be fast, as otherwise, high temperature leads to hydrolytic cleavage of the RNA chain [42]. Therefore, a conventional heating block is used, and the large volume has to be split to into smaller aliquots, which reach the high temperature faster. While this method leads to yield loss, due to significant transferring and handling of the sample, the alternative method takes several minutes to reach above 90 °C, where above mentioned hydrolytic cleavage will occur. Instead, we found that using a conventional microwave oven brings the sample to the boiling point in under 10 s, minimizing the time for magnesium-catalyzed hydrolysis and also omitting the need of changing reaction vessel. We found this to be faster and more robust than the described methods. However, we want to advise caution when working with closed containers in a microwave oven. As buffer exchange steps, purification and vessel changing reduce yield significantly, we utilize a one-pot procedure for both reaction steps that increase the final yield compared to prior purification or buffer-exchange while minimizing working hours and time spent in the laboratory. This is facilitated by using the two enzymes, T7 RNA polymerase and RNase H, from the same host organism, *E.coli*, allowing for highly similar reaction conditions. 

It has been found that *in vitro* transcribed RNA is immunogenic, due to 5′-triphosphate moieties and double-stranded side products [12]. With our protocol, both of these products are unlikely to occur, because the RNase H cleavage produces a 5′-monophosphate end, while a possible complementary RNA strand would not be cleaved down to short target units, due to lack of the appropriate cleavage guide. Furthermore, the produced 5′-monophosphate and 3′-hydroxyl residues can be seen as an advantage over methods utilizing ribozyme cleavage methods because of the 5′-hydroxyl and 2′,3′-cyclic phosphates produced [31], which is especially important for miRNA and siRNA activity [32].

In conclusion, our method proposed a new way of producing large amounts of single-stranded RNA at high yield. The obtained RNA sample is of high purity and has defined 5′-monophosphate and 3′-hydroxyl termini. Through the RNase H-catalyzed cleavage of a tandem transcript, the crude RNA product is of high purity which facilitates purification significantly.

### 3.2. Using Different Templates to Generate Tandem Transcripts

The template design is an essential step in the high yield of this protocol. Several different methods have been proposed, and advantages are discussed below. 

One of the methods described in the literature is rolling circle transcription to generate tandem repeats for RNase H cleavage in a few instances. The method described by Wang et al. [37] is analogous to the presented method by using rolling circle transcription from small ssDNA circles instead of plasmids encoding tandem repeats. However, we could not reproduce the results for our systems. 

Our strategy instead relies on well-established plasmid templates [39] that benefit from the specific T7 promoter followed by an optimal initiation site (5′ – GGG AGA – 3′) and are available in high yield at reasonable cost and effort, with the only limitation is the one time cost of creating the plasmid. Furthermore, the scale-up of plasmid reactions is simpler, due to the easy amplification in bacterial cultures [43]. Circularization of the plasmid insert is possible, which would allow for rolling circle transcription to be used on these constructs. However, the procedure is quite costly at rather a low yield (data not shown). An optimal number of repeats cannot be given at this point. Knowing the high processivity of T7RNAP, we suggest to incorporate the highest number of tandem repeats possible. Limitations here lie in the synthesis of such long repetitive fragments, as they are inaccessible to the techniques used for gene synthesis, such as Gibson assembly or gBlock synthesis [44]. Sequencing of plasmids encoding multiple repeats is a well-known problem in the field and apply here as well. However, the exact knowledge of how many repeats are present in the plasmid is not of relevance for the outcome of the method, and therefore, imperfect sequencing can be accepted to a certain degree.

We explored the use of ssDNA template encoding a lower number of repeats (2–5), which could be purchased cheaper and faster than an entire plasmid. However, we found that ssDNA strands complementary to the RNA target can lead to the complete degradation of the RNA in the presence of RNase H. This issue does not arise when using dsDNA templates. 

## 4. Materials and Methods

### 4.1. Plasmid Preparation

pUC19 plasmids have been purchased from GenScript with a solid-phase synthesized fragment inserted using PstI and SalI restriction site (vector and insert sequence can be found in the Supplementary Information). The plasmids have been amplified in *E.coli* NEB 5-alpha (NEB) and been purified using a Maxiprep purification kit (Machery-Nagel NucleoBond Xtra Maxi and QIAGEN Plasmid Maxi Kit). All plasmids have been linearized using BamHI (NEB) in the supplied reaction buffers at 37 °C for 1 h at a plasmid concentration of 20 ng/µL up to 500 µL total volume. To ensure complete linearization, reactions have been loaded on a 1% agarose gel in 0.5X TBE buffer (150 V, 20 min).

### 4.2. Gel Analysis

All polyacrylamide gels have been polymerized in 1X TBE (100 mM Tris, 90 mM boric acid, 1 mM EDTA) and 8 M urea, acrylamide:bisacrylamide 19:1 (Biorad, Solna, Sweden). Sample preparation: Typically, 1 µL sample was denatured in 9 µL loading solution (formamide, 240 µM bromophenol blue, 5 mM EDTA) and heated to 95 °C for 2 min. Gel specifics: 12 × 8 cm, Mini-Protean (Biorad, Solna, Sweden), run in 1X TBE buffer at 350 V for 1 h. Of the prepared loading solution of 10 µL, 1 µL was loaded. Small gels were post-stained with SYBR Gold (Invitrogen) and at 460 nm wavelength. 

Agarose gels have been prepared in 0.5X TBE and 1% agarose concentration and run in 0.5X TBE buffer for 150 V for 20 min. 

### 4.3. T7 In Vitro Transcription

Transcription reactions of construct 2 have been optimized in a small scale of 50 µl before large scale reactions. The same final conditions used for all constructs are shown in Supplementary Information Table S2 The input DNA has been used directly from the linearization reaction for transcription reaction without prior purification. Optimization reactions have been incubated for 1 h at 37 °C. All reagents have been prepared with nuclease-free water and sterile filtered before use. The work was conducted in an RNase-free environment, which was cleaned with RNase Zap (Invitrogen) and 95% ethanol. Large scale transcription reactions have been performed at 10 mL for 16 h at 37 °C and used as direct input for cleavage reaction without further processing. Transcription reactions for quantification experiments have been performed at 1 mL scale for 16 h at 37 °C.

### 4.4. RNase H Cleavage Reaction

Cleavage reactions from plasmid tandem transcription have been optimized for construct 2 in 20 µL volume, varying enzyme and cleavage guide concentration, as well as reaction time and temperature. The best conditions found have been used to cleave the large scale tandem transcription reaction in the same reaction tube. 

A chimeric cleavage guide usually consists of 4 central DNA nucleotides flanked by 4 2′-OMe modified nucleotides on each side. The sequences for each cleavage guide used and further discussion can be found in the Supplementary Information. For the cleavage reaction, 20% (v/v) of 100 µM cleavage guide (ordered from Integrated DNA technologies, IDT) has been added to the transcription reaction and heated in the microwave (Samsung MS23F301EAW) in a closed 50 mL centrifuge tube for 10 s and 5 s respectively at 450 W. For slow annealing, the mixture has been cooled down at 37 °C and room temperature for 15 min each. To start the reaction, *E.coli* RNase H has been added (in-house produced—2 µg/mL final concentration; Uniprot reference: P0A7Y4. NEB (catalog # M297L): 100 units/mL final concentration), as well as inorganic pyrophosphatase (IPPase) to 0.1 mg/mL. The cleavage reaction has been checked for completion on a 20% denaturing PAGE. Reactions took typically between 3 and 6 h for completion at 37 °C. In case of incomplete cleavage reactions, microwave annealing was repeated, and more RNase H was added. 

For simultaneous cleavage and transcription reactions, the reactions mixtures were prepared with the same volumes and concentrations as described above. Enzymes were added in the order: RNase H, IPPase, T7RNAP. Simultaneous large scale reactions were left for 16 h at 37 °C.

The reaction was stopped by adding an excess of EDTA at 100 mM and pH 6.5 (typically 30% (v/v) of 100 mM). Solutions were concentrated if necessary, and filtered before injection in HPLC with 22 µm PTFE or acetate cellulose syringe filters. 

### 4.5. Ion-Exchange HPLC Purification

For ion-exchange purification, a UltiMate 3000 UHPLC system (Thermo Scientific) with a DNAPAC^TM^ PA200 column of 22 × 250 mm by Thermo Scientific has been used (catalog # 088781). Running buffers were A: 20 mM sodium acetate, 20 mM sodium perchlorate, 10% acetonitrile, pH 6.5; B: 20 mM sodium acetate 600 mM sodium perchlorate, 10% acetonitrile, pH 6.5. Buffers have been filtered and degassed using 0.2 µm bottle top filters. The sequence was run at 5.5 mL/min flow rate and 75 °C to provide denaturing conditions. Gradient settings were: 0–7 min: 0% B; 7–16 min: 0–20% B; 16–46 min: Elution, typically 20–30% B; 46–62 min: 100% B; 62–73 min: 0% B. Specific elution gradients for constructs can be found in construct specification in the Supplementary Information. Fractions of interest have been analyzed on 20% PAGE gels according to paragraph 4.2. 

### 4.6. UV Spectroscopy

RNA concentrations have been determined using an Evolution 260 Bio UV-Visible Spectrophotometer (Thermo Scientific, Stockholm, Sweden). The obtained absorbance value at 260 nm has been used to calculate the RNA concentration according to the Lambert-Beer law

$$c=\frac{A}{\epsilon \times d}\times F_{dil}$$

where ϵ is the molar extinction coefficient, *d* is the path length and $F_{dil}$ is the sample’s dilution factor. ϵ and molar mass of RNA constructs has been calculated using the IDT oligo analyzer service (https://eu.idtdna.com/calc/analyzer/). 

### 4.7. Yield Calculation 

The yield Y was calculated as generally defined as the ratio of the obtained product and the limiting reagent. As for limiting reagent, the most abundant nucleotide in the target sequence was defined ($n_{in}$, obtained from concentration $c_{in}*V$, where V is the reaction volume). Since one equivalent of this nucleotide is consumed several times during the reaction, the nucleotide’s concentration was normalized to the nucleotide count ($N_{max}$) in the yield calculation.

$$Y=\;\frac{n_{out}}{\frac{c_{in}}{N_{max}}\times V}\times 100$$

As an example, construct 2 contains nine uridine residues, so the absolute UTP concentration was divided by 9. For the obtained product of 760 nmol from a 10 mL reaction, this gives

$$Y=\;\frac{760\;nmol}{\frac{3\;mM}{9}\times 10\;ml}\times 100=22.8\%$$

### 4.8. Quantification with Ion-Exchange HPLC 

RNA yields were quantified by comparison to an external HPLC standard curve with known amounts of construct 2. Standard curve samples of 1 mg, 0.5 mg, 0.25 mg, 0.125 mg and 0.062 mg (equals 149.8, 74.9, 37.4, 18.7 and 9.3 nmol) were prepared, and the concentration of the standard curve samples was determined with UV spectroscopy as described above.

All standard curve samples were brought to 1 mL with MilliQ-purified water and injected into HPLC as described in 4.5 with an elution gradient of buffer B of 20–30% over 30 min. The area under the curve (AUC) was calculated and used to create a standard curve which gave a slope of 30.8 nmol/mAU and an R^2^ value of 0.9997 (Supplementary Information Figure S1). 

To extract the molar amount of an injected target analyte sample $n_{T}$, the equation

$$n_{T}=\;\frac{AUC_{T}}{30.8}\times \frac{\epsilon_{c}}{\epsilon_{T}}$$

was used, where $AUC_{T}$ is the area under the curve for the analysed sample, $\epsilon_{c}$ the molar extinction coefficient of the standard curve sample and $\epsilon_{T}$ the molar extinction coefficient of the target analyte sample. Derivation of the equation and standard curve analytics can be found in the Supplementary Information.

## 5. Conclusions

Short RNA molecules are increasingly important as tools in basic research for downregulation or gene manipulation and are increasingly researched in structural biology to understand regulatory RNA mechanism. Through CRISPR/Cas and RNA interference, also the pharmaceutical industry requires large amounts of short RNAs with defined sequence and length. The protocol that we present here can provide both large amounts and high purity of especially these short RNAs. This addresses the needs of both basic research and pharmaceutical application for non-modified RNAs.

Described issues from 5′-triphosphate and double-stranded RNA side products are avoided as the RNA produced reliably carries a 5′-monophosphate and are single-stranded.

We expect that our method can make short RNAs more accessible to the scientific community, due to cheaper and simpler production and their results and application more valuable, due to the high reliability of the sample integrity.

## 6. Patents

A provisional patent application was filed at the UK patent office in March 2019. No decision has been taken to this point.

## Supplementary Materials

The following are available online https://www.mdpi.com/1420-3049/25/5/1142/s1, Sequences of plasmids, cleavage guides and target RNAs, Figure S1: Standard Curve HPLC, Table S1: Standard Curve HPLC, Table S2: Optimized parameters of single repeat IVTs, Table S3: Obtained yields from HPLC quantification, raw images of all gels.
