# Supplementary material for: One-Pot Production of RNA in High Yield and Purity Through Cleaving Tandem Transcripts
Source: Molecules. 2020 Mar 4;25(5):1142. doi: 10.3390/molecules25051142 (PMC7179201; doi:10.3390/molecules25051142)
Supplement: Supplementary file 1 [file molecules-25-01142-s001.pdf]

molecules

MDPI

Article

One-Pot production of RNA in high yield and purity through cleaving tandem transcripts

Hannes Feyrer [1], Raluca Munteanu [1], Lorenzo Baronti [1] and Katja Petzold [1,*]

[1] Department of Medical Biochemistry and Biophysics, Karolinska Institutet, Stockholm, 171 77 Sweden;
* Correspondence: katja.petzold@ki.se

Received: date; Accepted: date; Published: date

Supplementary information

Construct overview

RNA constructs and ordered DNA sequences. Cleavage guides were ordered at IDT at 100 nmol – 1 µmol scale with standard desalting purification. Chimeric cleavage guides contain mU instead of mT because of cheaper synthesis. All sequences are written in 5′ to 3′ direction.

Nucleotide key:
N: RNA
n: DNA
mN: 2′-OMe nucleotide

pUC19 vector backbone without insert:
gcgcccaatacgcaaaccgcctctccccgcgcgttggccgattcattaatgcagctggcacgacaggtttcccgactggaaagcgggcagtgag
cgcaacgcaattaatgtgagttagctcactcattaggcaccccaggctttacactttatgcttccggctcgtatgttgtgtggaattgtgagcggata
acaatttcacacaggaaacagctatgaccatgattacgccaagcttgcatgcctgcaggtcgactctagaggatccccgggtaccgagctcgaat
tcactggccgtcgttttacaacgtcgtgactgggaaaaccctggcgttacccaacttaatcgccttgcagcacatccccctttcgccagctggcgtaa
tagcgaagaggcccgcaccgatcgcccttcccaacagttgcgcagcctgaatggcgaatggcgcctgatgcggtattttctccttacgcatctgtg
cggtatttcacaccgcatatggtgcactctcagtacaatctgctctgatgccgcatagttaagccagccccgacacccgccaacacccgctgacgc
gccctgacgggcttgtctgctcccggcatccgcttacagacaagctgtgaccgtctccgggagctgcatgtgtcagaggttttcaccgtcatcacc
gaaacgcgcgagacgaaagggcctcgtgatacgcctatttttataggttaatgtcatgataataatggtttcttagacgtcaggtggcacttttcgg
ggaaatgtgcgcggaacccctatttgtttatttttctaaatacattcaaatatgtatccgctcatgagacaataaccctgataaatgcttcaataatatt
gaaaaaggaagagtatgagtattcaacatttccgtgtcgcccttattcccttttttgcggcattttgccttcctgtttttgctcacccagaaacgctggt
gaaagtaaaagatgctgaagatcagttgggtgcacgagtgggttacatcgaactggatctcaacagcggtaagatccttgagagttttcgcccc
gaagaacgttttccaatgatgagcacttttaaagttctgctatgtggcgcggtattatcccgtattgacgccgggcaagagcaactcggtcgccgc
atacactattctcagaatgacttggttgagtactcaccagtcacagaaaagcatcttacggatggcatgacagtaagagaattatgcagtgctgcc
ataaccatgagtgataacactgcggccaacttacttctgacaacgatcggaggaccgaaggagctaaccgcttttttgcacaacatgggggatca
tgtaactcgccttgatcgttgggaaccggagctgaatgaagccataccaaacgacgagcgtgacaccacgatgcctgtagcaatggcaacaacg
ttgcgcaaactattaactggcgaactacttactctagcttcccggcaacaattaatagactggatggaggcggataaagttgcaggaccacttctg
cgctcggcccttccggctggctggtttattgctgataaatctggagccggtgagcgtgggtctcgcggtatcattgcagcactggggccagatggt
aagccctcccgtatcgtagttatctacacgacggggagtcaggcaactatggatgaacgaaatagacagatcgctgagataggtgcctcactgat
taagcattggtaactgtcagaccaagtttactcatatatactttagattgatttaaaacttcatttttaatttaaaaggatctaggtgaagatcctttttga
taatctcatgaccaaaatcccttaacgtgagttttcgttccactgagcgtcagaccccgtagaaaagatcaaaggatcttcttgagatcctttttttctg
cgcgtaatctgctgcttgcaaacaaaaaaaccaccgctaccagcggtggtttgtttgccggatcaagagctaccaactctttttccgaaggtaactg
gcttcagcagagcgcagataccaaatactgtccttctagtgtagccgtagttaggccaccacttcaagaactctgtagcaccgcctacatacctcgc
tctgctaatcctgttaccagtggctgctgccagtggcgataagtcgtgtcttaccgggttggactcaagacgatagttaccggataaggcgcagcg

gtcgggctgaacggggggttcgtgcacacagcccagcttggagcgaacgacctacaccgaactgagatacctacagcgtgagctatgagaaa
gcgccacgcttcccgaagggagaaaggcggacaggtatccggtaagcggcagggtcggaacaggagagcgcacgagggagcttccaggg
ggaaacgcctggtatctttatagtcctgtcgggtttcgccacctctgacttgagcgtcgatttttgtgatgctcgtcaggggggcggagcctatgga
aaaacgccagcaacgcggcctttttacggttcctggccttttgctggccttttgctcacatgttctttcctgcgttatcccctgattctgtggataaccgt
attaccgcctttgagtgagctgataccgctcgccgcagccgaacgaccgagcgcagcgagtcagtgagcgaggaagcggaaga

Construct 1

Target RNA sequence (20 nt):
AGGGCCACAUCCCACUGCCA

Plasmid insert (27 copies):
taatacgactcactatagggagacactgccaagggccacatcccactgccaagggccacatcccactgccaagggccacatcccactgccaagg
gccacatcccactgccaagggccacatcccactgccaagggccacatcccactgccaagggccacatcccactgccaagggccacatcccactg
ccaagggccacatcccactgccaagggccacatcccactgccaagggccacatcccactgccaagggccacatcccactgccaagggccacatc
ccactgccaagggccacatcccactgccaagggccacatcccactgccaagggccacatcccactgccaagggccacatcccactgccaagggc
cacatcccactgccaagggccacatcccactgccaagggccacatcccactgccaagggccacatcccactgccaagggccacatcccactgcc
aagggccacatcccactgccaagggccacatcccactgccaagggccacatcccactgccaagggccacatcccactgccaagggccacatccc
actgccaagggccacatcccactgccaaggg

Cleavage guide:
mG mU mG mA c g g t mU mC mC mC

HPLC elution gradient:
18 – 26 %

Construct 2

Target RNA sequence (22 nt):
UGGCAGUGUCUUAGCUGGUUGU

Plasmid insert (26 copies):
taatacgactcactatagggagatggcagtgtcttagctggttgttggcagtgtcttagctggttgttggcagtgtcttagctggttgttggcagtgt
cttagctggttgttggcagtgtcttagctggttgttggcagtgtcttagctggttgttggcagtgtcttagctggttgttggcagtgtcttagctggttg
ttggcagtgtcttagctggttgttggcagtgtcttagctggttgttggcagtgtcttagctggttgttggcagtgtcttagctggttgttggcagtgtct
tagctggttgttggcagtgtcttagctggttgttggcagtgtcttagctggttgttggcagtgtcttagctggttgttggcagtgtcttagctggttgtt
ggcagtgtcttagctggttgttggcagtgtcttagctggttgttggcagtgtcttagctggttgttggcagtgtcttagctggttgttggcagtgtctt
agctggttgttggcagtgtcttagctggttgttggcagtgtcttagctggttgttggcagtgtcttagctggttgttggcagtgtcttagctggttgt

Cleavage guide:
mG mC mC mA a c a a mC mC mA mG

HPLC elution gradient:
20 – 30 %

Construct 3

Target RNA sequence (22 nt):
CAAUCAGCAAGUAUACUGCCCU

Plasmid insert (26 copies):
taatacgactcactatagggagacaatcagcaagtatactgccctcaatcagcaagtatactgccctcaatcagcaagtatactgccctcaatcagc
aagtatactgccctcaatcagcaagtatactgccctcaatcagcaagtatactgccctcaatcagcaagtatactgccctcaatcagcaagtatactg
ccctcaatcagcaagtatactgccctcaatcagcaagtatactgccctcaatcagcaagtatactgccctcaatcagcaagtatactgccctcaatca
gcaagtatactgccctcaatcagcaagtatactgccctcaatcagcaagtatactgccctcaatcagcaagtatactgccctcaatcagcaagtata
ctgccctcaatcagcaagtatactgccctcaatcagcaagtatactgccctcaatcagcaagtatactgccctcaatcagcaagtatactgccctcaa
tcagcaagtatactgccctcaatcagcaagtatactgccctcaatcagcaagtatactgccctcaatcagcaagtatactgccctcaatcagcaagt
atactgccctcaatcagcaagtatactgccctc

Cleavage guide:
mA mU mU mG a g g g mC mA mG mU

HPLC elution gradient:
20 – 30 %

Construct 4

Target RNA sequence (22 nt):
CCGGCCGCCUGCGGCACUGCCU

Plasmid insert (26 copies):
taatacgactcactatagggagaccggccgcctgcggcactgcctccggccgcctgcggcactgcctccggccgcctgcggcactgcctccggc
cgcctgcggcactgcctccggccgcctgcggcactgcctccggccgcctgcggcactgcctccggccgcctgcggcactgcctccggccgcctg
cggcactgcctccggccgcctgcggcactgcctccggccgcctgcggcactgcctccggccgcctgcggcactgcctccggccgcctgcggcac
tgcctccggccgcctgcggcactgcctccggccgcctgcggcactgcctccggccgcctgcggcactgcctccggccgcctgcggcactgcctcc
ggccgcctgcggcactgcctccggccgcctgcggcactgcctccggccgcctgcggcactgcctccggccgcctgcggcactgcctccggccgc
ctgcggcactgcctccggccgcctgcggcactgcctccggccgcctgcggcactgcctccggccgcctgcggcactgcctccggccgcctgcgg
cactgcctccggccgcctgcggcactgcct

Cleavage guide:
mG mG mC mC mG mG A G G C mA mG mU mG mC mC

HPLC elution gradient:
20 – 30 %

Construct 5

Target RNA sequence (22 nt):
AACCAGCUAAGACACUGCCAGG

Plasmid insert (26 copies):
taatacgactcactatagggagaaaccagctaagacactgccaggaaccagctaagacactgccaggaaccagctaagacactgccaggaacc
agctaagacactgccaggaaccagctaagacactgccaggaaccagctaagacactgccaggaaccagctaagacactgccaggaaccagct
aagacactgccaggaaccagctaagacactgccaggaaccagctaagacactgccaggaaccagctaagacactgccaggaaccagctaaga
cactgccaggaaccagctaagacactgccaggaaccagctaagacactgccaggaaccagctaagacactgccaggaaccagctaagacact
gccaggaaccagctaagacactgccaggaaccagctaagacactgccaggaaccagctaagacactgccaggaaccagctaagacactgcca
ggaaccagctaagacactgccaggaaccagctaagacactgccaggaaccagctaagacactgccaggaaccagctaagacactgccagga
accagctaagacactgccaggaaccagctaagacactgccagg

Cleavage guide:
mG mG mU mU c c t g mG mC mA mG

HPLC elution gradient:
20 – 30 %

Construct 6

Target RNA sequence (75 nt):
CGUCACCCUCCUCAAGUAUACUUCAAAGGACAUUUAACUAAAACCCCUACGCAUUUAU
AUAGAGGAGACAAGUCG

Plasmid insert (5 copies):
taatacgactcactatagggagaacaagtcgcgtcaccctcctcaagtatacttcaaaggacatttaactaaaacccctacgcatttatatagagga
gacaagtcgcgtcaccctcctcaagtatacttcaaaggacatttaactaaaacccctacgcatttatatagaggagacaagtcgcgtcaccctcctc
aagtatacttcaaaggacatttaactaaaacccctacgcatttatatagaggagacaagtcgcgtcaccctcctcaagtatacttcaaaggacattta
actaaaacccctacgcatttatatagaggagacaagtcgcgtcaccctcctcaagtatacttcaaaggacatttaactaaaacccctacgcatttata
tagaggagacaagtcgcgtcaccctcctcaagtatacttcaaaggacatttaactaaaacccctacgcatttatatagaggagacaagtcgcgtca
ccctcctcaagtatacttcaaaggacatttaactaaaacccctacgcatttatatagaggagacaagtcgcgtc

Cleavage guide:
mG mA mC mG c g a c mU mU mG mU

HPLC elution gradient:
30 – 40 %

Quantification of target RNA using a control standard curve

A control standard curve using 149.8, 74.9, 37.4, 18.7 and 9.3 nmol of construct 2 was created. The area under the curve was plotted against the injected amount of RNA. A linear regression (Figure SI 1) gave the equation

$$AUC_c = 30.8 * n_c$$

This can be used to determine the amount of another RNA construct by determining the area under curve of any RNA x assuming area under the curve is proportional to the injected amount of sample times the molar extinction coefficient according to the Lambert-Beer law.

$$AUC_x = \varepsilon_x * n_x$$

Having a constant flow rate and path length allows to compare between a control sample c (the standard curve) and a target sample t:

$$\frac{AUC_c}{AUC_t} = \frac{\varepsilon_c * n_c}{\varepsilon_t * n_t}$$

Solving this equation for n_t allows us to apply the slope obtained from the standard curve shown in the first equation to determine the amount of substance of a target sample:

$$n_t = \frac{AUC_t * \varepsilon_c * n_c}{\varepsilon_t * AUC_c} = \frac{AUC_t}{30.8} * \frac{\varepsilon_c}{\varepsilon_t}$$

In plain words, we are extracting the n_t from the slope of the standard curve and account for the different ε due to sequence and size differences.

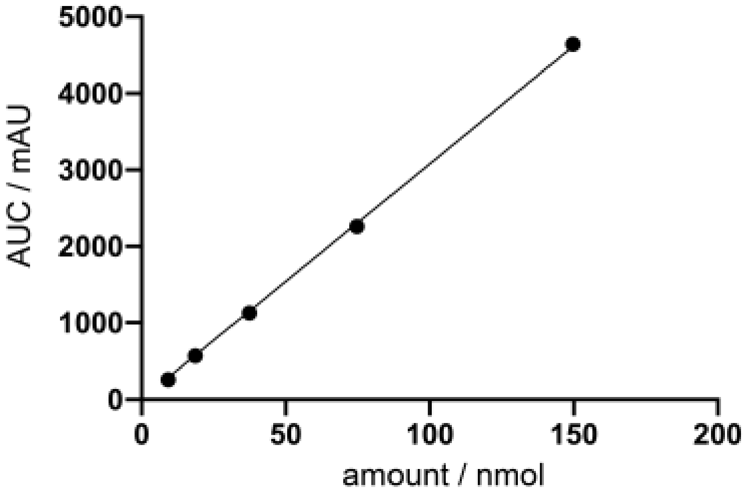

Figure SI 1: Obtained standard curve and linear regression through the data points. The regression was forced through the origin and gave y = 30.8 x.

Table SI 1. Samples of construct 2 for HPLC standard curve and obtained area under the curve (AUC)

m/ mg	n / nmol	AUC / mAU
0.062	9.2	261
0.125	18.7	570
0.25	37.4	1127
0.5	74.9	2260
1	149.7	4641

IVT from single-repeat templates

Templates encoding a single construct repeat are single stranded and only double-stranded in the promoter sequence. The template strands carry two 2′-OMe modifications on the 5′-end, to increase 3′-homogeneity of the transcript. The shorter T7 promoter sequence was annealed to the template by heating to 95 °C at 25 μM concentration, and cooling down on to room temperature.

Sequences:
n: DNA
mN: 2′-OMe RNA

T7 promoter: t t a a t a c g a c t c a c t a t a
Construct 1*: mU mU g c a g t g g g a t g t g g c c c t t a t a g t g a g t c g t a t t a a
Construct 2*: mA mC a a c c a g c t a a g a c a c t g c c a t a t a g t g a g t c g t a t t a a

Table SI 2. Optimized reaction conditions used for T7 IVT from single repeat ssDNA templates

Reagent	Tandem transcripts	Construct 1*	Construct 2*
Tris-Cl pH 8.0	100 mM	100 mM	80 mM
$MgCl_2$	10 mM	30 mM	40 mM
Dithiothreitol	10 mM	3 mM	6 mM
Spermidine	20 mM	2 mM	2 mM
NMP (GMP/AMP/UMP)	5 mM	10 mM	24 mM
NTPs (each)	3 mM	3 mM	3 mM (ATP: 1 mM)
DNA template	2 ng/μl	500 nM	500 nM
T7 RNA polymerase	0.3 mg/ml	0.2 mg/ml	0.3 mg/ml
Inorganic pyrophosphatase	0.1 mg/ml	0.1 mg/ml	0.1 mg/ml
DMSO	-	4 % (v/v)	20 % (v/v)

Obtained yields from HPLC quantification

Table SI 3. Obtained yields for different constructs from HPLC quantification in % as described above

1*	1	2*	2	3	4	5	6
3.77	23.85	2.1	22.8	39.15	14.45	11.2	28.3
	17.91		17.6	14.7	4.53	7	18.8
			17.33				

Original raw images of all gels (red box marks shown region)

This image cannot currently be displayed.

Figure 2A

This image cannot currently be displayed.

Figure 2B

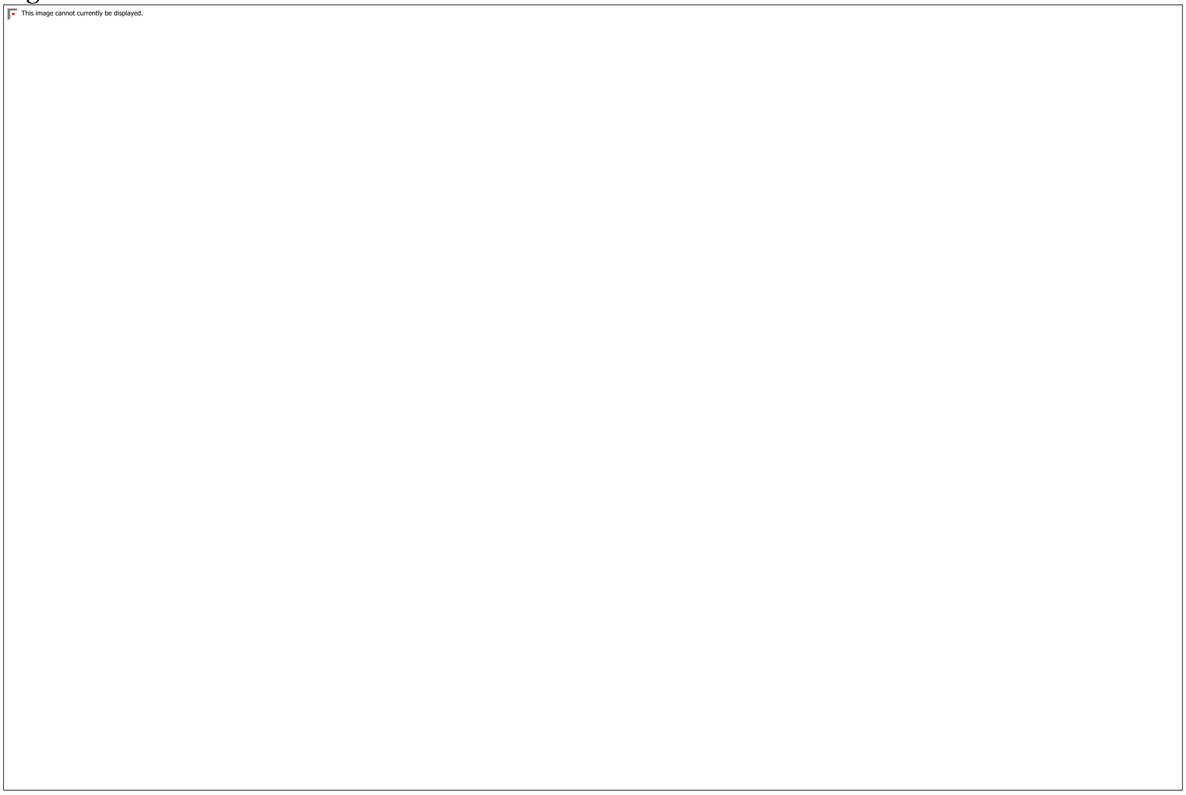

Figure 2C

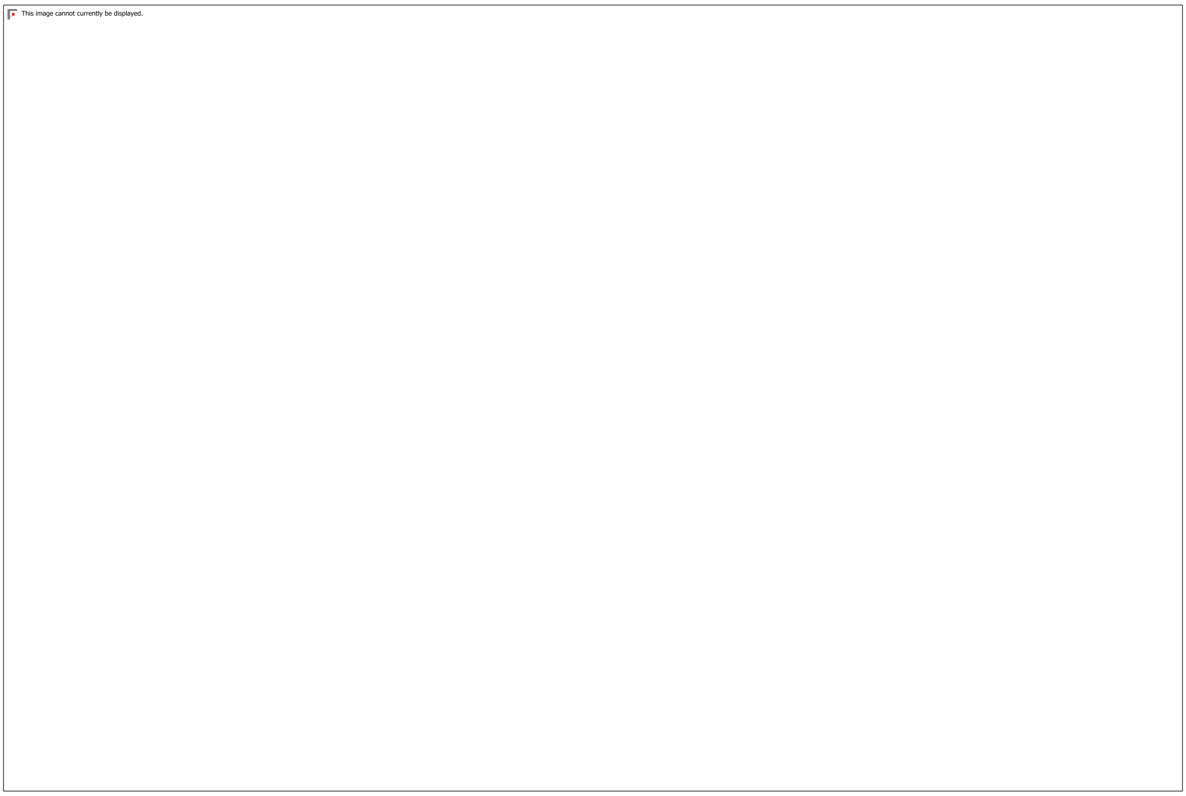

Figure 3A

© 2020 by the authors. Submitted for possible open access publication under the terms and conditions of the Creative Commons Attribution (CC BY) license (http://creativecommons.org/licenses/by/4.0/).
